# Preinvasive nonhost resistance of *Arabidopsis* against melanized appressorium-mediated entry of multiple nonadapted *Colletotrichum* fungi

**DOI:** 10.1080/15592324.2021.2018218

**Published:** 2022-01-03

**Authors:** Hiroki Irieda

**Affiliations:** Academic Assembly, Institute of Agriculture, Shinshu University, Nagano, Japan

**Keywords:** Phytopathogenic fungi, melanized appressorium-mediated entry, nonhost resistance, preinvasive defense, *Arabidopsis*, *Colletotrichum*, PEN2

## Abstract

Nonhost plants effectively block a vast number of nonadapted fungal pathogens at the preinvasive stage. On the host plants, adapted fungal pathogens such as *Colletotrichum* species invade into plant epidermal cell by penetration peg developed from melanized appressorium, followed by invasive hyphal extension. I reported nonadapted *Colletotrichum* fungi that showed an increased rate of melanized appressorium-mediated entry (MAE) into the *pen2* mutant of nonhost *Arabidopsis thaliana* (hereafter *Arabidopsis*). It was also found that other MAE-type nonadapted *Colletotrichum* fungi with no penetration into the *pen2* mutant invaded *Arabidopsis* in the presence of additional mutations such as *edr1*, *gsh1*, *eds5*, *cas*, and *chup1* in the *pen2* background. Thus, many immune components contribute to the preinvasive nonhost resistance (NHR) of *Arabidopsis* against *Colletotrichum* MAE, and PEN2-related defense takes priority over other defense pathways. Here, I show that among the above nonadapted fungi, *Colletotrichum nymphaeae* PL1-1-b exhibited relatively lower incompatibility with the nonhost *Arabidopsis* with increased MAE in each single mutant of *edr1*, *gsh1*, *eds5*, and *cas*, although other nonadapted fungi almost never invaded these single mutants. Based on the relationships between *Colletotrichum* MAE and the *Arabidopsis* immune-related components, *Colletotrichum-Arabidopsis* incompatibility and multilayered immunity in the preinvasive NHR of *Arabidopsis* are discussed in this study.

Fungal pathogens directly invade the plant epidermis, followed by the development of invasive hyphae inside; therefore, penetration into epidermal cells is the most crucial step for successful infection. Only a few adapted fungal pathogens can infect a specific plant (defined as the host). If the plant is a nonhost, a robust and broad-spectrum defense termed nonhost resistance (NHR) effectively prevents the invasion of a vast number of nonadapted fungi in incompatible interactions.^1,2^

Incompatible interactions between the model brassicaceous plant *Arabidopsis thaliana* (hereafter *Arabidopsis*) and nonadapted powdery mildew fungi, such as *Blumeria graminis* f. sp. *hordei*, have been well studied for NHR. Publications reported that *pen1, pen2, pen3, pmr4, ataf1, pldδ, cam7, cyp81F2*, and *vps9a* mutant plants allowed increased entry of these obligate biotrophs into the epidermis.^3–11^ PENETRATION 1 (PEN1) syntaxin mediates a secretory pathway to the fungal penetration site.^3,12^ PENETRATION 2 (PEN2) myrosinase, CYTOCHROME P450 FAMILY 81 SUBFAMILY F POLYPEPTIDE 2 (CYP81F2) monooxygenase, and PENETRATION 3 (PEN3) transporter are involved in metabolism and transport of tryptophan-derived secondary metabolites,^5,6,8,13,14^ while CALMODULIN 7 (CaM7) is a Ca^2+^ sensor which regulates PEN3 function.^10^ POWDERY MILDEW RESISTANT 4 (PMR4) callose synthase functions in wound and papillary callose formation.^4^ ARABIDOPSIS THALIANA ACTIVATING FACTOR 1 (ATAF1) transcription factor suppresses abscisic acid biosynthesis and signaling.^7^ PHOSPHOLIPASE Dδ (PLDδ) catalyzes phosphatidic acid production on the membrane in stress responses.^9^ VACUOLAR PROTEIN SORTING 9a (VPS9a) guanine-nucleotide exchange factor is required for the delivery of membrane material to the haustorial encasement.^11^ Thus, many pathways and components including as-yet-unknown factors elaborately underpin the *Arabidopsis* NHR against powdery mildew fungi.

The hemibiotroph *Colletotrichum* fungus was also instrumental in the research on NHR in *Arabidopsis*. Of those mentioned above, *pen1, pen2, pen3, pmr4*, and *cyp81F2* mutants were used for the fungal entry test with nonadapted *Colletotrichum tropicale* S9275, a mulberry isolate, and the contribution of PEN2, PEN3, and CYP81F2 to preinvasive defense were demonstrated.^15,16^ Hiruma et al. also performed screening of *Arabidopsis* mutants, and ENHANCED DISEASE RESISTANCE 1 (EDR1) and γ-GLUTAMYLCYSTEINE SYNTHETASE 1 (GSH1) were found as NHR contributors against entry of *C. tropicale* S9275 to the epidermis.^16,17^ EDR1 protein kinase mediates the induction of antifungal plant defensins.^16,18^ GSH1 is involved in the generation of PEN2-related metabolites through glutathione biosynthesis, while GSH1 is also required for postinvasive defense.^13,19^ Immune kinases BRASSINOSTEROID INSENSITIVE 1-ASSOCIATED RECEPTOR KINASE 1 (BAK1), BOTRYTIS-INDUCED KINASE 1 (BIK1), and AVRPPHB SUSCEPTIBLE1 (PBS1)-LIKE 1 (PBL1) working in the defense signaling triggered by pathogen-associated molecular patterns were also involved in preinvasive NHR against *C. tropicale* S9275.^20^ On all these mutant plants, however, *C. tropicale* S9275 could barely develop invasive hyphae through melanized appressorium, a typical infection structure of *Colletotrichum* fungi. Instead, this pathogen successfully entered the *Arabidopsis* epidermis when the carbohydrate-induced hyphal tip-based entry (HTE).^15–17,20^ HTE, only reported in *C. tropicale* S9275 at present, was not associated with appressorium formation, thereby suggesting an atypical infection mode with different morphogenesis after perception of carbohydrate nutrients released from the wounded sites of the plants.^15^ Therefore, there is little knowledge on *Arabidopsis* NHR against melanized appressorium-mediated entry (MAE) into the epidermis by *Colletotrichum* fungi. This is in contrast to those against hemibiotroph *Pyricularia oryzae* (Syn. *Magnaporthe oryzae*), which also shows MAE during plant infection.^21–26^ In *P. oryzae-Arabidopsis* incompatible interaction, PEN2 functions as a NHR contributor against MAE and *P. oryzae* slightly invaded *pen2* mutant.^21^ Furthermore, many other immune components such as ARABIDOPSIS G-PROTEIN β-SUBUNIT 1 (AGB1), POWDERY MILDEW RESISTANT 5 (PMR5), MILDEW RESISTANCE LOCUS O 2 (MLO2), MAP KINASE 6 (MPK6), ERECTA, SUPPRESSOR of BAK1-INTERACTING RECEPTOR-LIKE KINASE 1 (SOBIR1), and EXTRA-LARGE G PROTEIN 2 (XLG2), the single mutation of which did not affect the breakdown of penetration resistance, showed decreased preinvasive NHR in the *pen2* background.^21–26^

I have recently reported three nonadapted *Colletotrichum* fungi, *C. nymphaeae* PL1-1-b (MAFF240037), *C. fioriniae* CC1 (MAFF306550), and *C. siamense* MAF1 (MAFF243010), isolates from Japanese flowering cherry, cosmos, and apple, respectively, which all showed increased MAE into the *pen2* mutant.^27^ It was also found that two nonadapted *Colletotrichum* fungi, *C. siamense* COC4 (MAFF243696) and *C. orbiculare* 104-T (MAFF240422), isolates from hau tree and cucumber, respectively, could not invade *pen2* single mutant, but was able to invade on *pen2* mutant with additional mutations such as *edr1, gsh1, eds5, cas*, and *chup1*.^27^ The MAE rates of *C. fioriniae* CC1 and *C. siamense* MAF1 into *pen2* mutants were also increased in the presence of *edr1, gsh1, eds5, cas*, and *chup1*.^27^ ENHANCED DISEASE SUSCEPTIBILITY 5 (EDS5) is a MATE family transporter that is required for the transport of the salicylic acid precursor outside the chloroplasts.^28,29^ CALCIUM-SENSING RECEPTOR (CAS) is a Ca^2+^-sensing receptor involved in transient Ca^2+^ signaling in chloroplasts during plant immunity.^30^ CHLOROPLAST UNUSUAL POSITIONING 1 (CHUP1) is a regulator of epidermal chloroplast response, a newly discovered immune-related response.^27^ These findings reveal that PEN2, EDR1, GSH1, EDS5, CAS, and CHUP1 all contribute to preinvasive NHR of *Arabidopsis* against *Colletotrichum* MAE. It is noteworthy that the potential effects of *edr1, gsh1, eds5, cas*, and *chup1* mutations on the MAE of *C. fioriniae* CC1 and *C. siamense* MAF1 were actualized on the *pen2* background.^27^ Furthermore, quantitative real-time PCR analysis showed that many defense-related genes were induced after inoculation of *C. fioriniae* CC1 only in *pen2* among all tested single mutants.^27^ These results indicate that the PEN2-related pathway functions as a higher-layer preinvasive defense and takes priority over other defense pathways, at least in preinvasive NHR against these nonadapted MAE-type *Colletotrichum* fungi.

Intriguingly, among the above MAE-type nonadapted fungi (Figure 1a), *C. nymphaeae* PL1-1-b could partly achieve MAE in the epidermis of wild-type *Arabidopsis* Col-0, and the *chup1* single mutant permitted an increase in the trend of the MAE rate^27^ (Figure 1b and 1c). These results suggest the potential ability of *C. nymphaeae* PL1-1-b to overcome the higher-layer preinvasive defense(s) of *Arabidopsis* to a certain degree, although the PEN2-related defense pathway is still working against this pathogen^27^ (Figure 1c). To clarify this point, the MAE rate of *C. nymphaeae* PL1-1-b into *edr1, gsh1, eds5*, and *cas* single mutants was quantified, and it was found that this pathogen showed a significant increase or increasing trend in the MAE rate in all single mutants compared to that in Col-0 (Figure 1c). These results indicate that *C. nymphaeae* PL1-1-b partly overcomes the higher-layer preinvasive defense(s) in *Arabidopsis* and that each immune pathway related to EDR1, GSH1, EDS5, or CAS is effective against this pathogen in the wild-type plant. Thus, *C. nymphaeae* PL1-1-b is not adapted to *Arabidopsis*, but its incompatibility with the nonhost *Arabidopsis* is relatively low.

**Figure 1.**
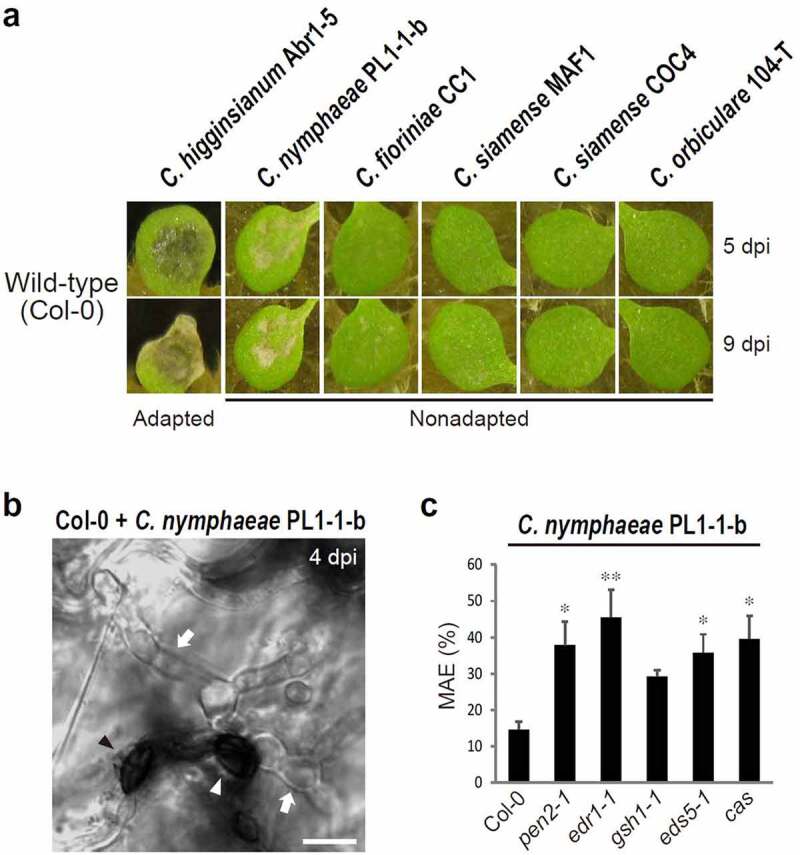
Nonadapted *C. nymphaeae* PL1-1-b exhibited relatively lower incompatibility with the nonhost *Arabidopsis* and showed increased MAE into *edr1, gsh1, eds5*, and *cas* single mutants. (a) Pathogenicity of *Colletotrichum* fungi on wild-type *Arabidopsis* (Col-0). A conidial suspension of adapted *C. higginsianum* Abr1-5 and nonadapted *C. nymphaeae* PL1-1-b, *C. fioriniae* CC1, *C. siamense* MAF1, COC4, and *C. orbiculare* 104-T was inoculated onto cotyledons of Col-0 and incubated. The photographs were taken at 5 and 9 days post-inoculation (dpi). (b) Invasion of *C. nymphaeae* PL1-1-b into epidermis of Col-0 at 4 dpi. The white arrowhead and arrows indicate melanized appressorium and invasive hyphae, respectively. The black arrowhead represents melanized appressorium with no invasive hypha. Scale bar, 10 µm. (c) MAE rate of *C. nymphaeae* PL1-1-b into *Arabidopsis* mutants at 4 dpi. For quantification of MAE, 300 melanized appressoria were investigated. The means and SE were calculated from six independent plants. The asterisks indicate significant difference from the control (Col-0) (*P < .05, **P < .01, one-way ANOVA with Dunnett’s test).

To visualize the relationships between *Colletotrichum-Arabidopsis* incompatibility and immune components in *Arabidopsis* preinvasive NHR, the *Colletotrichum* MAE rates were integrated from a previous study^27^ and newly obtained data, although I did not quantified the entry rates of *C. nymphaeae* PL1-1-b on *Arabidopsis* with multiple mutations, because the effect of each mutation was already actualized in the corresponding single mutants (Figure 2). Consistent with the results of the pathogenicity assay of *Colletotrichum* fungi on the wild-type *Arabidopsis* Col-0 (Figure 1a), the MAE rates of five nonadapted fungi were notably lower than those of the adapted fungus *C. higginsianum* Abr1-5 (MAFF305635), a *Brassicaceae* pathogen (Figure 2). Five nonadapted *Colletotrichum* strains displayed differential ability to overcome preinvasive NHR in *Arabidopsis* mutants in the following order: *C. nymphaeae* PL1-1-b > *C. fioriniae* CC1 > *C. siamense* MAF1 > *C. siamense* COC4 > *C. orbiculare* 104-T. *C. nymphaeae, C. fioriniae*, and *C. siamense* are classified into *Colletotrichum* clades with broad host range (acutatum or gloeosporioides). Thus, these nonadapted strains might exhibit lower incompatibility compared to *C. orbiculare* 104-T which generally shows narrow host range. Since I used one representative strain from one species, except *C. siamense*, it is difficult to confirm that the *Arabidopsis* response is specific to the fungal species. On the other hand, the interaction of each nonadapted strain with the nonhost *Arabidopsis* provided a clear insight into preinvasive NHR in *Arabidopsis*. To discuss the general interactions between fungal species and *Arabidopsis*, it is important to evaluate the interaction of multiple fungal strains from one species with *Arabidopsis*.

**Figure 2.**
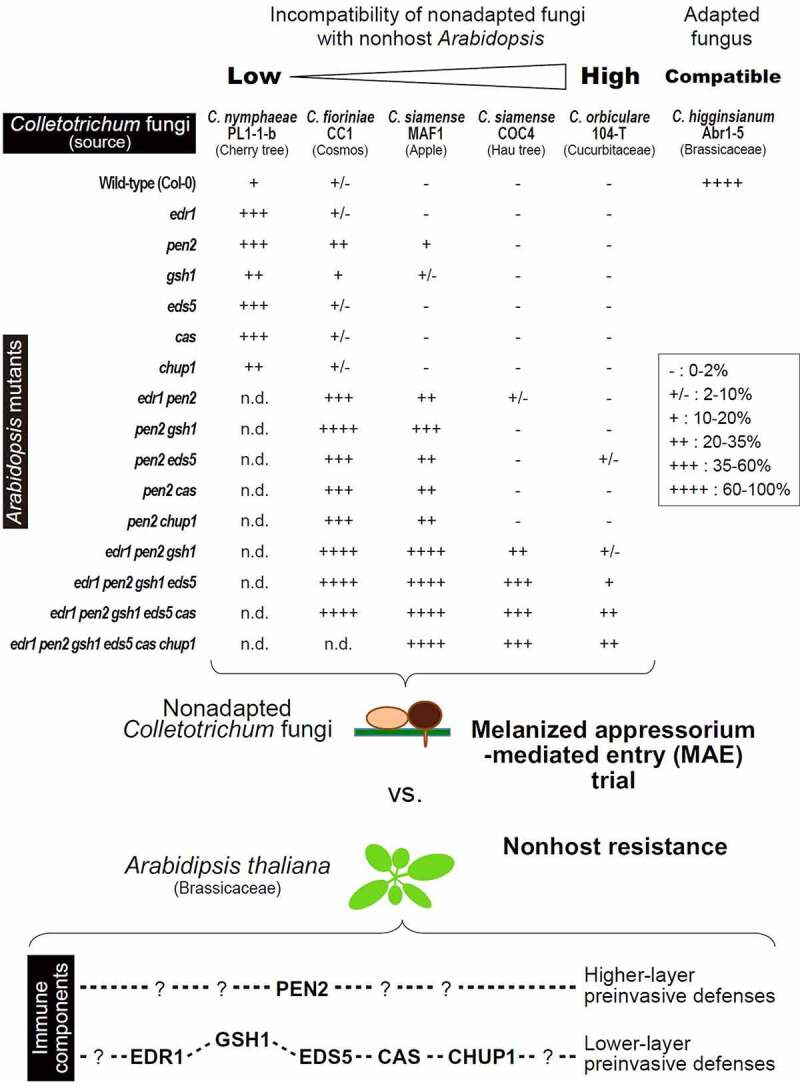
Relationships between MAE of nonadapted *Colletotrichum* fungi and preinvasive NHR in *Arabodipsis thaliana*. Invasion abilities of nonadapted *C. nymphaeae* PL1-1-b, *C. fioriniae* CC1, *C. siamense* MAF1, COC4, and *C. orbiculare* 104-T into nonhost *Arabidopsis* mutants were evaluated based on the MAE rates and classified. Adapted *C. higginsianum* Abr1-5 was showed as a control. PEN2-related defense takes priority over other immune components. The percentage of fungal entry test as “-”, “+/-”, “+”, “++”, “+++”, and “++++” was 0–2%, 2–10%, 10–20%, 20–35%, 35–60%, and 60–100%, respectively. n.d.: not determined.

Apart from the *pen2* single mutant, the *gsh1* single mutant showed a slight reduction in the preinvasive NHR against *C. fioriniae* CC1 and *C. siamense* MAF1 compared to other single mutants^27^ (Figure 2). It was previously reported that GSH1 and PEN2 contribute to the same pathway in preinvasion NHR against HTE of *C. tropicale* S9275.^17^ The slight effect of *gsh1* single mutation against the MAE of these two fungi might partly reflect the link to the PEN2 pathway. GSH1 was of paramount importance for the resistance against fungal entry of *C. fioriniae* CC1, *C. siamense* MAF1, and *C. siamense* COC4 in the *pen2* background^27^ (Figure 2); however, in the case of *C. nymphaeae* PL1-1-b, the *gsh1* single mutant did not show a higher MAE rate than the other single mutants, suggesting that the GSH1-related pathway is not very effective against *C. nymphaeae* PL1-1-b (Figure 1c).

Although PEN2, EDR1, GSH1, EDS5, CAS, and CHUP1 contribute to preinvasive NHR of *Arabidopsis* against *Colletotrichum* MAE, the preinvasive NHR of the *edr1 pen2 gsh1 eds5 cas chup1* hexatruple mutant against *C. orbiculare* 104-T still has sufficient reserves^27^ (Figure 2). It is important to identify additional immune pathways that are effective against *C. orbiculare* 104-T and other untested nonadapted *Colletotrichum* strains that are highly incompatible with nonhost *Arabidopsis*.

PEN2 is a core NHR contributor to many fungal pathogens, including MAE-type *P. oryzae* and atypical HTE-type *C. tropicale* S9275.^5,8,13,15,21^ Additionally, PEN2 conferred NHR against typical MAE-type *Colletotrichum* fungi^27^ (Figure 2). The use of nonadapted *Colletotrichum* fungi that can invade a single mutant, like *pen2*, through MAE, is promising; this will accelerate future research on NHR underlying *Colletotrichum-Arabidopsis* incompatible interactions. Currently, research on screening for NHR contributors against MAE of *C. fioriniae* CC1 is underway. In particular, the identification of immune component(s) other than PEN2, working in higher-layer preinvasive defenses is essential. *C. nymphaeae* PL1-1-b may avoid or suppress the function of such immune component(s) to partly overcome preinvasive NHR in the nonhost *Arabidopsis*.
